# Changing Trends in Eye-Related Complaints Presenting to the Emergency Department in Beirut, Lebanon, over 15 Years

**DOI:** 10.1155/2018/4739865

**Published:** 2018-03-13

**Authors:** Haytham I. Salti, Carl-Joe Mehanna, Bachir Abiad, Nicola Ghazi, Samih Raad, Anita Barikian, Randa Haddad, Adnan Ashkar, Elie Harmouche, Elie Zaghrini, Afif Mufarrij

**Affiliations:** ^1^Department of Ophthalmology, American University of Beirut Medical Center, Beirut, Lebanon; ^2^Department of Ophthalmology, University of Texas Southwestern Medical Center, Dallas, TX, USA; ^3^Department of Ophthalmology, Cleveland Clinic Foundation, Abu Dhabi, UAE; ^4^Department of Internal Medicine, Indiana University School of Medicine, Indianapolis, IN, USA; ^5^Department of Ophthalmology, University of Tennessee Health Science Center, Hamilton Eye Institute, Memphis, TN, USA; ^6^American University of Beirut Medical Center, Beirut, Lebanon; ^7^Department of Emergency Medicine, Henry Ford Hospital, Detroit, MI, USA; ^8^Department of Emergency Medicine, Lebanese American University Medical Center-Rizk Hospital, Beirut, Lebanon; ^9^Department of Emergency Medicine, American University of Beirut Medical Center, Beirut, Lebanon

## Abstract

**Objective:**

To report the 15-year trend in ophthalmic presentations to the emergency department (ED) at the only medical center in Lebanon that provides 24-hour ophthalmologic care.

**Methods:**

Retrospective review of 1967 patients presenting to the ED with eye-related complaints between September 1997 and August 1998 and between September 2012 and August 2013. Diagnoses were classified into 4 categories according to the International Society of Ocular Trauma and include penetrating eye injuries, nonpenetrating eye trauma, nontraumatic ophthalmic emergencies, and nontraumatic, nonurgent ophthalmic conditions.

**Results:**

One thousand sixty eye-related presentations out of 39,158 total ED visits (2.71%) presented in 1997 compared to 907 out of 46,363 in 2012 (1.96%). Penetrating and nonpenetrating eye emergencies decreased between 1997 and 2012 (7.17% to 4.19%, *p* = 0.003 and 52.64% to 29.00%, *p* < 0.001, resp.) while nonurgent cases increased from 30.19% to 53.47% (*p* < 0.001). 57% of patients were covered by third-party guarantors in 1997 versus 73% in 2012.

**Conclusion:**

Our results demonstrate a significant increase in nonurgent cases in parallel with the proportion of third-party payers, an issue to be addressed by public health policies and proper resource allocation. A detailed nationwide review is needed to make solid recommendations for the management of ophthalmologic presentations in the ED.

## 1. Introduction

Ophthalmologic complaints constitute around 1–6% of total emergency department (ED) visits [1]. ED utilization varies depending on multiple factors such as ease of access to an ED, patient education, and financial coverage—all related to the local healthcare policies—a particularity to each country. For instance, in industrialized nations, ED visits are covered primarily by governmental agencies or third-party private insurance companies, while in developing countries, it is usually the patient who has to fund the costs of the visit out of pocket. This discrepancy in financial coverage helps explain on the one hand the high proportion of nonurgent, noninjury-related ophthalmic complaints presenting to the ED in industrialized countries [2–4] and on the other hand, the significant number of serious injury-related eye conditions constituting the majority of ED presentations in developing countries [5, 6]. Furthermore, the lack of patient awareness of and abidance by eye-safety measures seems to also play an important role in the increasing number of serious ocular injuries, especially in developing countries [7–9].

Lebanon is a particular case of a “hybrid healthcare system,” falling between the two extremes. Emergency outpatient presentations can be either covered by third-party privately owned insurance companies or self-financed; in fact, while there are several government agencies that cover in-patient hospitalizations (Ministry of Public Health, National Social Security Fund), private insurance companies are the only ones that cover certain outpatient visits, including the ED. Thus, while third-party guarantors are growing in their healthcare role, covering a gradually increasing number of patients presenting to the ED, a sizeable proportion of patients are still paying out of pocket [10]. Of note as well is patients' awareness and education that are still overall suboptimal, yet government-sponsored campaigns have been more effective over the past few years and might be contributing to a decline in serious injuries [11].

Our aim in this study is to report the changing trends over a span of 15 years in ophthalmologic-related presentations to the ED at the American University of Beirut Medical Center (AUBMC), which is the only tertiary medical center in Lebanon that provides 24-hour ophthalmologic care.

## 2. Materials and Methods

### 2.1. Patient Data Retrieval

After obtaining approval from the AUBMC Institutional Review Board and in agreement with the tenets of the Declaration of Helsinki, a retrospective chart review of patients presenting to the ED with eye-related complaints was conducted. Charts of patients who presented to the ED between September 1997 and August 1998 and between September 2012 and August 2013 were collected from the medical record department of the hospital and were included if both the chief complaint and final diagnosis were related to any of the visual, orbital, or periorbital systems. After deidentification, the following data was retrieved: patient age, gender, presentation, final diagnosis, management, and mode of financial coverage. A comparison was done between the two periods to elucidate the trend over time.

### 2.2. Classification of Emergency Presentations

Emergency presentations were classified by one of the authors (BA) into broad categories according to their final diagnosis using the International Classifications of Diseases, Ninth Revision (ICD-9) coding system. This scheme was adapted from the classification of the International Society of Ocular Trauma [12] and represents a comparable approach to that used in similar studies [13, 14]. Briefly, we divided conditions into four broad categories based on their seriousness and acuteness: penetrating eye injuries, nonpenetrating eye trauma, nontraumatic ophthalmic emergencies, and nontraumatic, nonurgent ophthalmic conditions, that is, where vision is not threatened. The last category's presentations were further stratified into the following subcategories for more detailed analysis: subconjunctival hemorrhage, conjunctivitis, keratitis, eyelid-related disorders, and contact lens-related disorders.  Table 1 provides a list of ICD-9 diagnoses that fit these criteria.

### 2.3. Statistical Analysis

Clinical data obtained were analyzed using IBM SPSS v.23. Descriptive statistics were reported as mean and standard deviations for continuous variables and as numbers and percentages for categorical variables. The Wilcoxon signed-rank test was used to compare means. A *p* value <0.05 was considered statistically significant.

## 3. Results

A total of 39,158 visits were made to the ED at AUBMC during 1997 compared to 46,363 in 2012. Eye- and ocular adnexa-related presentations comprised 1060 (2.71%) and 907 (1.96%), respectively. The characteristics of these two populations are shown in Table 2. The two groups were statistically comparable with regard to age, but there was a significant increase in the percentage of women presenting to the ED in 2012 as compared to 1997 (49.61% versus 32.74%, respectively, *p* < 0.001). In both groups, penetrating eye injuries were the highest among patients aged between 31 and 45 years (Table 3).

With regard to the cause for presentation, there was a statistically significant decrease in the percentage of penetrating (7.17% to 4.19%, *p* = 0.003) and nonpenetrating (52.64% to 29.00%, *p* < 0.001) eye injuries. In parallel, there was a clear increase in the nontraumatic presentations to the ED from 40.19% in 1997 to 66.81% in 2012, *p* < 0.001. In particular, nonurgent cases increased from 30.19% to 53.47% (*p* < 0.001) of all ophthalmic presentations between the 2 periods. Detailed analysis reveals an overall proportional increase across the board of all “nonurgent” ophthalmologic presentations between the 2 periods, except for contact lens-related issues whose percentage among nonurgent ophthalmologic presentations per each time period increased by almost 50% (Table 4).

**Figure 1:**
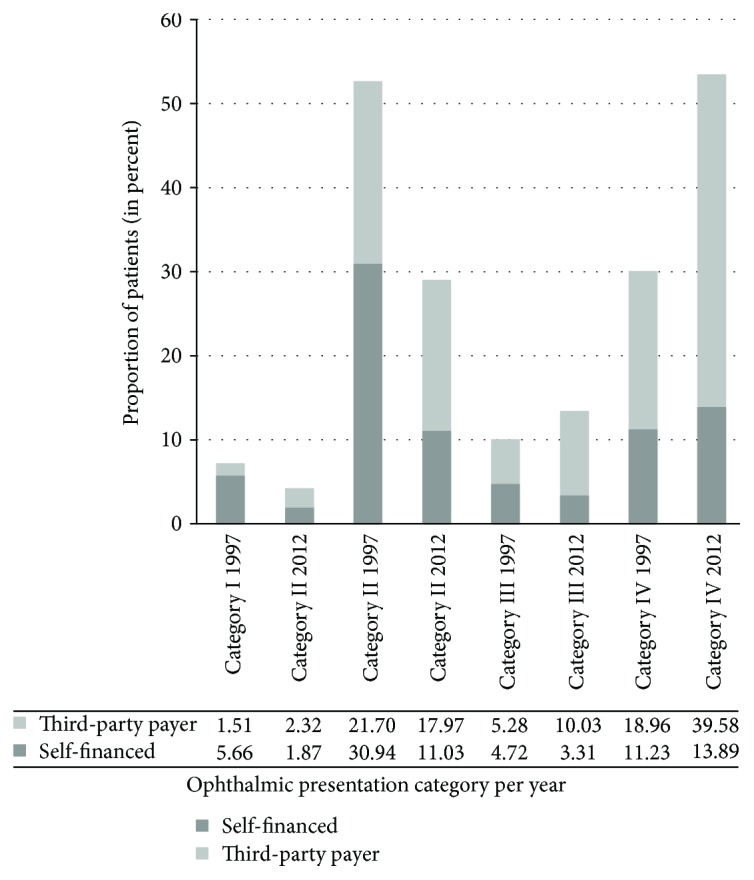
Percentage of financial coverage per ophthalmic presentation category for 1997 and 2012. Category I: penetrating eye injuries; category II: nonpenetrating eye trauma; category III: nontraumatic ophthalmic emergencies; category IV: nontraumatic, nonurgent ophthalmic conditions.

Looking at financial coverage, 57% of these patients were covered by third-party guarantors in 1997 versus 73% in 2012. A breakdown of financial coverage by category is presented in Figure 1.

## 4. Discussion

This is the first study looking at ED trends of eye-related complaints in Lebanon. Epidemiological data on ED presentations from the Eastern Mediterranean region are rare and none examined trends over time [5, 6, 15].

Results reveal that patients presenting with penetrating eye injuries decrease over time. This may be attributed to the public health campaigns developed for road and construction site safety both popularized in the years between 1997 and 2012 [16]. In fact, injuries from motor vehicle accidents have decreased substantially despite the rise in the number of cars and motorcycles in Lebanon [17].

In parallel, there is a clear increase in ED presentations of nonurgent cases as was demonstrated in recently published studies [4, 18]. This is potentially explained by the robust increase in third-party payers' financial coverage of health issues for the Lebanese population in general [19] and patients with complaints presenting to the ED in particular. In fact, the lenient and user-friendly policies of third-party guarantors have contributed to facilitating the ED visit, a phenomenon recognized and demonstrated in the US by Channa et al. [4]. Figure 1 demonstrates the growing proportion of third-party payers across all categories and particularly the nonurgent cases. An in-depth analysis of these presentations, especially from the latter years, reveals an important portion to be related to contact lens wear. The most important proportion, however, is that of the disorders of the conjunctiva and eyelids—conditions that can be managed in an outpatient setting, thereby avoiding the cost and burden of the ED utilization.

Efforts should therefore be invested in referring these patients to outpatient clinics and community health centers for management and care. From studies such as this, one can recommend healthcare policy amendment and resource redistribution restricting the ED resources to urgent cases, thus offering several advantages: patients seen during working hours in an eye clinic would benefit from a comprehensive examination including screening for potentially blinding conditions such as glaucoma, diabetic retinopathy, and age-related macular degeneration. Also, resources in the ED could be better focused on patients who truly need urgent care. Furthermore, since ED visit costs significantly more than an office examination, national health care costs could potentially be greatly reduced.

While important on a health policy level, our study has several limitations that ought to be addressed. First, like most retrospective chart reviews, it is limited to the data entered and available at the time of the ED presentation. Second, our population coverage and representation might be influenced by AUBMC being the only 24-hour center offering ophthalmologic care. Finally, a longer follow-up might give a clearer picture of the trend observed.

In summary, our study opens a window on the changing trends of eye complaints of patients presenting to the ED, an issue that should be tackled by public health policymakers with an emphasis on proper utilization of the current resources. While revealing, such work should be duplicated on a nationwide level with emphasis on a longer follow-up period looking at a more detailed review to come up with solid recommendations for eye-related complaints presenting to the ED.

## Figures and Tables

**Table 1:** International society of ocular trauma classification of ocular trauma, including selected diagnoses.

Category	Examples of ICD-9 diagnoses
Penetrating eye injuries	871—open wound of eyeball, including laceration, rupture, avulsion, and penetration of eye

Nonpenetrating eye trauma	921—contusion of eye and adnexa

Nontraumatic ophthalmic emergencies	360—endophthalmitis
	361.0—retinal detachment with retinal defects
	362.31—central retinal artery occlusion
	362.35—central retinal vein occlusion
	365.22—acute angle-closure glaucoma
	376.01—orbital cellulitis
	377.0—papilledema
	377.3—optic neuritis
	940—burn confined to the eye and adnexa (including chemical burns)

Nontraumatic, nonurgent ophthalmic conditions	372—disorders of the conjunctiva, including acute conjunctivitis, subconjunctival hemorrhage
	373—inflammation of eyelids

**Table 2:** Characteristics of patients presenting to the ED with ophthalmic complaints in 1997 compared to 2012.

	1997	2012
Total number of ED visits	39,158	46,363
Eye-related ED visits	1060 (2.71%)	907 (1.96%)
Average patient age (years)	31.4	30.8
Gender		
Male	713 (67.26%)	457 (50.39%)
Female	347 (32.74%)	450 (49.61%)
Penetrating eye injuries	76 (7.17%)	38 (4.19%)
Nonpenetrating eye trauma	558 (52.64%)	263 (29.00%)
Nontraumatic ophthalic emergencies	106 (10.00%)	121 (13.34%)
Nontraumatic, nonurgent ophthalmic conditions	320 (30.19%)	485 (53.47%)

**Table 3:** Percent distribution of ophthalmic complaints among different age groups in 1997 compared to 2012.

Age group (years)	Penetrating eye injuries		Nonpenetrating eye trauma		Nontraumatic ophthalmic emergencies		Nontraumatic nonurgent ophthalmic conditions	
	1997	2012	1997	2012	1997	2012	1997	2012
0–5	3.1	1.5	60.9	53	76.6	75.37	23.4	24.62
6–16	1.9	5.6	58.5	56.07	77.4	74.77	22.6	25.23
17–30	1.3	4.89	63.9	32.33	81.7	62.4	18.3	37.6
31–45	4	5.73	64.6	40.1	79.4	66.15	20.6	33.85
>45	2.3	2.78	42.5	31.48	69.7	61.57	30.3	38.43

**Table 4:** Breakdown and percentage of nonurgent ophthalmologic presentations between 1997 and 2012.

	1997	2012
Subconjunctival hemorrhage	92 (28.75%)	133 (27.42%)
Conjunctivitis	108 (33.75%)	153 (31.75%)
Keratitis	20 (6.25%)	32 (6.60%)
Eyelid-related disorders	61 (19.06%)	79 (16.29%)
Contact lens-related disorders	39 (12.19%)	87 (17.94%)
Total	320 (100%)	485 (100%)
